# Transcriptome Analysis Reveals the Differentially Expressed Genes Associated with Growth in Guangxi Partridge Chickens

**DOI:** 10.3390/genes13050798

**Published:** 2022-04-29

**Authors:** Minghui Shao, Kai Shi, Qian Zhao, Ying Duan, Yangyang Shen, Jinjie Tian, Kun He, Dongfeng Li, Minli Yu, Yangqing Lu, Yanfei Tang, Chungang Feng

**Affiliations:** 1 College of Animal Science and Technology, Nanjing Agricultural University, Nanjing 210095, China; 2019105007@stu.njau.edu.cn (M.S.); 2020205003@stu.njau.edu.cn (K.S.); zhaoqian0117@gmail.com (Q.Z.); 2020105006@stu.njau.edu.cn (Y.D.); 2019205005@njau.edu.cn (Y.S.); 2019805091@njau.edu.cn (J.T.); 2019105006@njau.edu.cn (K.H.); lidongfeng@njau.edu.cn (D.L.); yuminli@njau.edu.cn (M.Y.); 2 State Key Laboratory for Conservation and Utilization of Subtropical Agro-Bioresources, College of Animal Science and Technology, Guangxi University, Nanning 530004, China; lyq@gxu.edu.cn; 3 Guangxi Fufeng Agricultural and Animal Husbandry Group Co., Ltd., Nanning 530024, China; 18853857810@163.com

**Keywords:** Guangxi Partridge chicken, growth rate, breeding, transcriptome, hypothalamus, pituitary, muscle, liver

## Abstract

The Guangxi Partridge chicken is a well-known chicken breed in southern China with good meat quality, which has been bred as a meat breed to satisfy the increased demand of consumers. Compared with line D whose body weight is maintained at the average of the unselected group, the growth rate and weight of the selected chicken group (line S) increased significantly after breeding for four generations. Herein, transcriptome analysis was performed to identify pivotal genes and signal pathways of selective breeding that contributed to potential mechanisms of growth and development under artificial selection pressure. The average body weight of line S chickens was 1.724 kg at 90 d of age, which showed a significant increase at 90 d of age than line D chickens (1.509 kg), although only the internal organ ratios of lung and kidney changed after standardizing by body weight. The myofiber area and myofiber density of thigh muscles were affected by selection to a greater extent than that of breast muscle. We identified 51, 210, 31, 388, and 100 differentially expressed genes (DEGs) in the hypothalamus, pituitary, breast muscle, thigh muscle, and liver between the two lines, respectively. Several key genes were identified in the hypothalamus-pituitary-muscle axis, such as *FST*, *THSB*, *PTPRJ*, *CD36*, *PITX1*, *PITX2*, *AMPD1*, *PRKAB1*, *PRKAB2*, and related genes for muscle development, which were attached to the cytokine–cytokine receptor interaction signaling pathway, the PPAR signaling pathway, and lipid metabolism. However, signaling molecular pathways and the cell community showed that elevated activity in the liver of line S fowl was mainly involved in focal adhesion, ECM-receptor interaction, cell adhesion molecules, and signal transduction. Collectively, muscle development, lipid metabolism, and several signaling pathways played crucial roles in the improving growth performance of Guangxi Partridge chickens under artificial selection for growth rate. These results support further study of the adaptation of birds under selective pressure.

## 1. Introduction

Indigenous Chinese chicken breeds are distinct from commercial breeds of fowl due to their excellent production traits, such as great meat quality and disease resistance, and they possess a large share of the poultry market. Unlike developed breeds, however, native birds barely achieved their growth potential [1]. To meet the increased command for meat products, genetic selection for more yield in indigenous populations is essential. Selective pressure on broilers contributes to greater yield of cut parts, such as breasts and thighs [2].

Skeletal muscle is the largest organ of the body and, in addition to its role in motion, muscle also functions as the endocrine and metabolic organ to regulate energy balance, glucose uptake, and metabolic activities [3]. Meanwhile, quantity and quality of skeletal muscle are important characters of yield and as factors of interest by breeders and consumers. In recent years, increased studies have investigated the genetic mechanism that affects growth performance or meat quality in different breeds or developmental stages. Several genes (e.g., *MYH15*, *MYOZ2*, *MYBPC3*, *IGF2*, *BCL-2*, *JUN*, and *FOS*) were identified in breast muscle of fast-growing fowl breeds, which were involved in muscle regulation, muscle construction, and myoblast differentiation [4]. Differentially expressed lncRNAs between slow-growing native Gushi chickens and fast-growing Arbor Acres chickens influenced muscle development and growth [5]. *SREBF1*, *GHR*, and *FASN* have been recognized widely as causal genes of meat quality, and they were distinctively expressed in cattle muscle with diverse meat quality and growth performance [6]. Genome-wide association study revealed that *MLNR*, *MED4*, *CAB39L*, *LDB2*, and *IGF2BP1* may be potential candidate gens for chicken growth and carcass traits [7].

Hormones of the endocrine system are important regulators in muscle development, of which growth hormone (GH), thyroid hormone, and androgens have been researched commonly in many species [8]. The secretion of hormones is primarily regulated by the dual stimulatory and inhibitory of the hypothalamus and pituitary [9]. The hypothalamic neurons also secrete orexigenic and anorexia neuropeptides, such as AGRP and α-MSH, to influence appetite and food intake [10]. Transcriptome analysis of the hypothalamus screened several genes in male Ross 308 strains with different growth rates, such as *POMC*, *NMU*, *NPW*, *PMCH*, *GAL*, and *FOS*, which displayed different expression patterns [11]. A total of 39 differentially expressed genes (DEGs) were identified by transcriptome analysis between high egg-yielding and low egg-yielding hens, and these DEGs were involved mainly in metabolism and transport of amino acids [12].

In addition, the liver similarly plays an important role in controlling energy homeostasis and metabolic activity. Increased growth may result in increased organ workload, increased metabolism, and metabolic diseases, such as cardiovascular ailments and ascites, which may lead to the failure of body systems [13]. Changes in gene expression and metabolic activity in the liver were correlated significantly with lipid metabolism through the PPAR signaling pathway and steroid biosynthesis [14]. Transcriptome analysis of meat birds, layer strains, and a F1 hybrids showed that the *FoxOs* was correlated highly with body weight through regulation of glucose metabolism [15].

Up to now, researchers have focused primarily on explaining discrepant production performances in low- and high-performing individuals in the same population or in different breeds, and they rarely explore effects on growth under generational selective pressure. The Guangxi Partridge chicken is a well-known indigenous chicken breed in southern China with good meat quality. Selective breeding has been used in Guangxi Partridge chicken to produce two lines with high selection intensity. Line S has been under selection for higher growth rate for four generations, while line D under selection for egg number. These two chicken lines with different growth performance were employed to explore the effect of short-term selection. In the present study, transcriptome sequencing was adopted to investigate the expression profile of mRNAs in the hypothalamus, pituitary, breast muscle, thigh muscle, and liver between two chicken lines that had different selection goals. Identification and functional analysis of differentially expressed mRNA indicated several important pathways that may be involved in rapid growth, development, and metabolism.

## 2. Materials and Methods

### 2.1. Experimental Animals and Tissue Samples

Breeding lines S and D of Guangxi Partridge chicken were raised at the Guangxi Fufeng Agricultural and Animal Husbandry Group Co., Ltd. (Nanning, China). Lines S and D have been selected for four generations from the same population of native Guangxi Partridge chicken. In line S, the higher body weight is the main selection goal, and selection proportion is about 0.03 for the male and about 0.20 for the female. In line D, the higher egg production is the main selection goal, under the premise of maintaining the body weight not less than the mean value of its initial population. Hybrid chickens generally take 90 to 130 d to reach market weight. Ten cockerels were selected randomly from two lines at 90 d of age. Body weight and organ weights (heart, liver, spleen, lung, kidney, and testis) of each individual were measured separately after fasting for 12 h (10 individual chickens per line). Then, the hypothalamus, pituitary, liver, breast muscle, and thigh muscle tissues were collected immediately after the birds were sacrificed. The dissected tissues were placed quickly into microtubes, which were frozen rapidly in liquid nitrogen and then preserved at −80 °C.

### 2.2. Examination of Muscle Fibers

Three chickens in each group were selected for measurement of muscle fiber size and density. Tissues of breast muscle and thigh muscle were dehydrated by gradient alcohol, paraffin embedded, sectioned, and stained terminally with hematoxylin and eosin. Tissue sections were observed using a CX31 microscope (Olympus Corporation, Tokyo, Japan) at a magnification of 200×. For each sample, three images without tissue damage were analyzed by Image-J (National Institute of Mental Health, Bethesda, MD, USA). Then, muscle fiber area and fiber density were calculated.

### 2.3. RNA Extraction and Sequencing

The hypothalamus, pituitary, liver, breast muscle, and thigh muscle tissues from three chickens from each group were used for RNA sequencing. Total RNA was extracted from each sample using Trizol reagent (Invitrogen, Carlsbad, CA, USA). The purity of RNA was measured by a Nanodrop 2000 spectrophotometer (Thermo Scientific, Waltham, MA, USA). Then, an Agilent 2100 Bioanalyzer (Agilent Technologies, Palo Alto, CA, USA) and RNase free agarose gel electrophoresis were used to detect RNA integrity and quality. RNA integrity number (RIN), which ranged from 0 to 10, reflects the quality of RNA. The RIN values of all RNA samples in this study were > 8.

mRNA was enriched with Oligo (dT) beads, and rRNA was removed using a Ribo-ZeroTM Magnetic Kit (Epicentre, Madison, WI, USA). The enriched mRNA was fragmented using fragmentation buffer, and then they were reversed into cDNA. RNase H, dNTP, DNA polymerase I, and buffer were used to synthesize the second-strand cDNA. Then, the cDNA fragments were purified, end repaired, poly (A) added, and ligated to Illumina sequencing adapters. The ligation products were size selected by agarose gel electrophoresis, PCR amplified. Paired-end sequencing was performed on Illumina Nova-Seq 6000 sequencing platform by Gene Denovo Biotechnology Co. (Guangzhou, China).

### 2.4. Transcriptome Mapping and Assembly

Reads obtained from the sequencing machines were further filtered by fastp (v 0.18.0). The reads that contained adapters or > 10% of unknown nucleotides (N) and low-quality reads that contained > 50% of low quality (Q-value ≤ 20) bases were filtered. Clean reads were then mapped to the chicken reference genome GCF_000002315.6 using HISAT (v2. 2.4). Then, the mapped reads were assembled by using StringTie (v1.3.1). FPKM (fragment per kilobase of transcript per million mapped reads) values were calculated to quantify the expression abundance of transcript.

### 2.5. RNA-seq Data Analysis

Differential expression analysis of RNA of the two groups was performed using the DESeq2 R package (v1.32.0). The transcripts with the parameters of adjusted *p*-value < 0.05 and |log2 (FoldChange)| > 1 were considered as DEGs. GO of the DEGs was performed with the online software DAVID (https://david.ncifcrf.gov/summary.jsp, accessed on 3 September 2021). Kyoto Encyclopedia of Genes and Genomes (KEGG) pathway analysis was performed by the R package ClusterProfiler (v4.0.5). The protein-protein interaction network was constructed based on DEGs using the online STRING (v11.5, https://cn.string-db.org, accessed on 8 October 2021) database and was visualized using Cyto-scape software (v3.8.2). The statistical power of this study was estimated by the SSPA R package (v2.22.1) [16].

### 2.6. Validation of RNA Expression by Quantitative-PCR

Nine genes were selected from the DEG of breast muscle and thigh muscle for quantitative verification. The primers were designed using Primer3 (v.0.4.0). qRT-PCR was determined using the Step-One plus Real-Time PCR System with ChamQ Universal SYBR qPCR Master Mix (#Q441-02, Vazyme, Nanjing, China). The information on primers is listed in Supplementary Table S1. The 20 μL reaction mixture contained 10 μL 2× ChamQ Universal SYBR qPCR Master Mix, 0.4 μL forward primer, 0.4 μL reverse primer, 2 μL cDNA, and 7.2 μL ddH_2_O. The procedure was as follows: 30 s of pre-denaturation at 95, followed by 40 cycles of 95 °C for 10 s and 60 °C for 30 s, solubility curve period at 95 °C for 15 s, 60 °C for 1 min, and 95 °C for 15 s. Expression of all genes was normalized to the *GAPDH* level. The relative mRNA expression levels were calculated using the normalized relative quantification method followed by 2^−ΔΔCT^.

### 2.7. Statistical Analysis

Data were analyzed using Prism (v.6.0; GraphPad Software, San Diego, CA, USA) for Student’s *t*-test. Data were expressed as the mean ± standard deviation (SD). When the *p* value was <0.05, the results were considered statistically significant. The linear regression and correlation of gene expression between RNA-seq and qRT-PCR were calculated by Excel.

## 3. Results

### 3.1. Growth Performance and Differences in Muscle Fiber between the Two Lines

**Figure 1:**
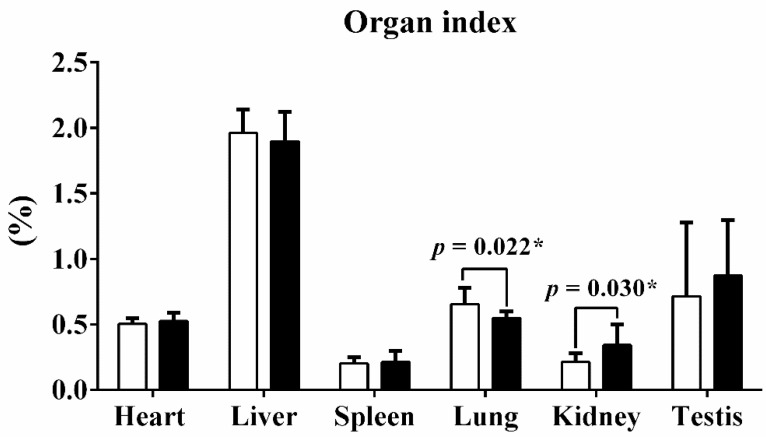
The organ index of six tissues between two different lines. The organ index quantified the ratio of organ weight to total body weight. Values are shown as mean ± SD (*n* = 10 individuals per group). * represents *p* < 0.05.

Guangxi Partridge chickens of lines D and S were raised for 90 d from hatching, and the weights of the body, heart, liver, lungs, spleen, kidneys, and testes were recorded after harvest. The mean weight of line S chickens was 1.724 ± 0.128 kg, which was significantly heavier than line D birds at 1.509 ± 0.084 kg (*p* < 0.001). Mass of heart, liver, and kidneys of line S chickens also increased (Table 1). The organ index quantified the ratio of organ weight to total body weight. Only the lung index and kidney index exhibited obvious alterations after being calibrated with body weight, but the other organ indices remained stable (Figure 1).

**Figure 2:**
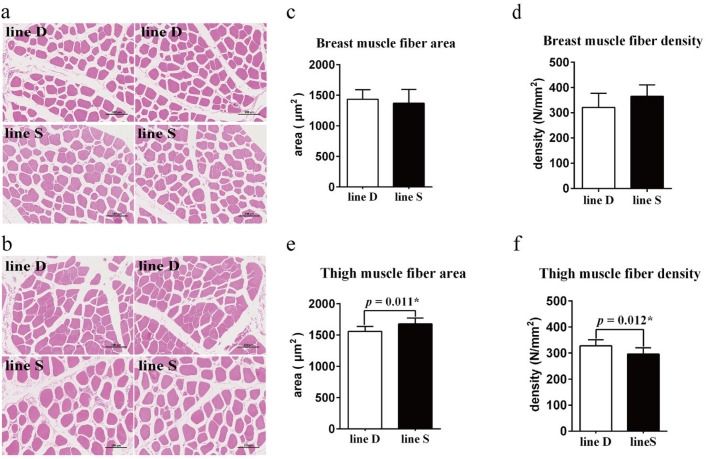
Comparison of muscle fiber morphology and myofiber characteristics between the two lines. Myofiber morphology observation of breast muscle (**a**) and thigh muscle (**b**) tissue section. There are two images for each chicken line of different muscle tissue. Scale bar, 100 µm. Muscle fiber area (**c**) and muscle fiber density (**d**) of breast muscle. Muscle fiber area (**e**) and muscle fiber density (**f**) of thigh muscle. Values are shown as mean ± SD (*n* = 3 per group). * represents *p* < 0.05.

Muscle fiber morphology between the two chicken lines appeared similar (Figure 2a,b), and the difference in myofiber area and myofiber density (count of myofibers per mm^2^ of a cross-sectional area) in breast muscle between the two lines was not significant (*p* > 0.05) (Figure 2c,d). However, the myofiber area was enhanced significantly, and there was a lower quantity of myofiber of thigh muscle in line S (*p* < 0.05) (Figure 2e,f).

### 3.2. Analysis of Differentially Expressed Genes

**Figure 3:**
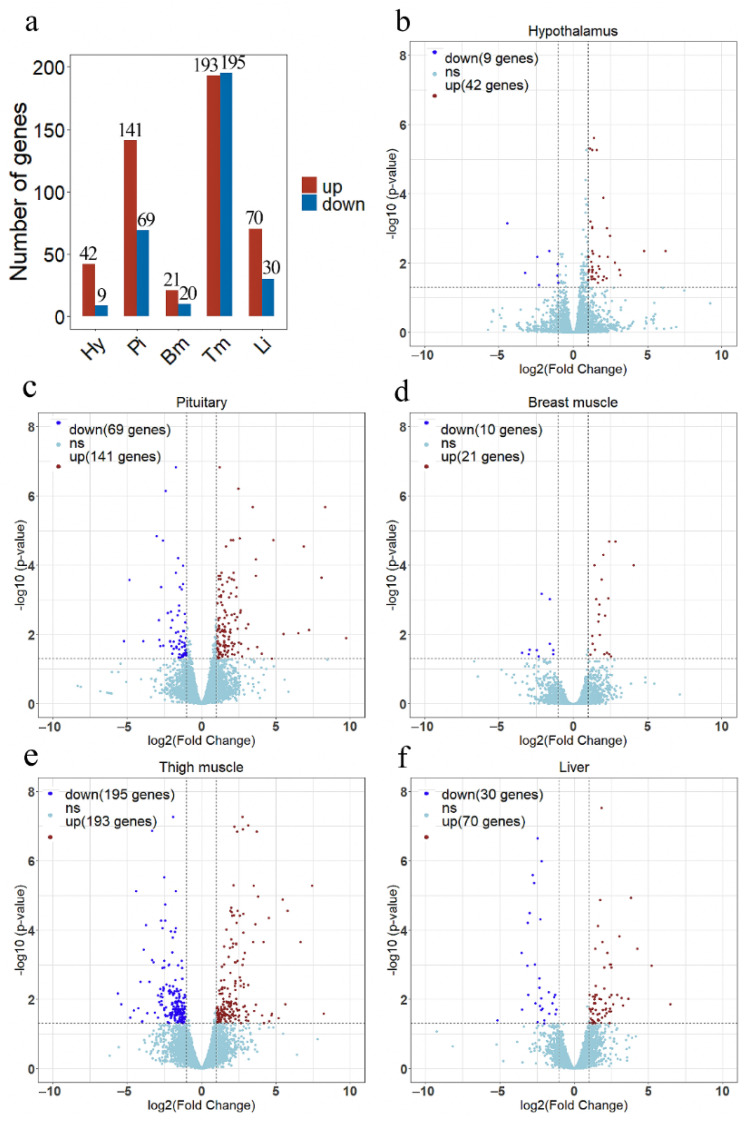
The results of differential expression analysis in each tissue between line S and line D. (**a**) The overview of the number of differentially expressed genes (DEGs) in each tissue. The volcano plots show differentially expressed genes in the hypothalamus (**b**), pituitary (**c**), breast muscle (**d**), thigh muscle (**e**), and liver (**f**). Red dots indicate up-regulated genes in line S, dark blue dots indicate down-regulated genes in line S, and the light blue dots indicate no significant changes in gene expression. Significant DEGs were identified by filtering the *p*-value < 0.05 and |log_2_ (Fold Change)| > 1. Hy, Hypothalamus; Pi, Pituitary; Bm, Breast muscle; Tm, Thigh muscle; and Li, Liver.

In each line, three animals were selected with close to average body weight, and five different tissues were collected from each sample. 30 RNA samples ware used to construct separate RNA-seq libraries. A total 110 Gb of raw data were obtained from RNA sequencing. More than 90% of the clean reads were mapped to the broiler reference genome (GCF_000002315.6), which included 87% in the exonic regions and 9% in the introns. The average percentages of Q20 and Q30 bases were >97.34% and >92.75%, respectively (Supplementary Table S2). There was an average of 50,244,659 clean reads for each sample after quality control. Correlation analysis of gene expression levels showed a high correlation among samples, which indicated that the selection of experimental samples was reliable (Supplementary Figure S1). The statistical power ranged from 0.69 to 0.84 for different tissues (Supplementary Figure S2). A total of 51, 210, 31, 388, and 100 DEGs were identified in the hypothalamus, pituitary, breast muscle, thigh muscle, and liver between lines S and D (Figure 3a). Heatmap of the DEGs showed that these DEGs easily differentiated line S from line D (Supplementary Figure S3).

#### 3.2.1. Hypothalamus and Pituitary Tissues

**Figure 4:**
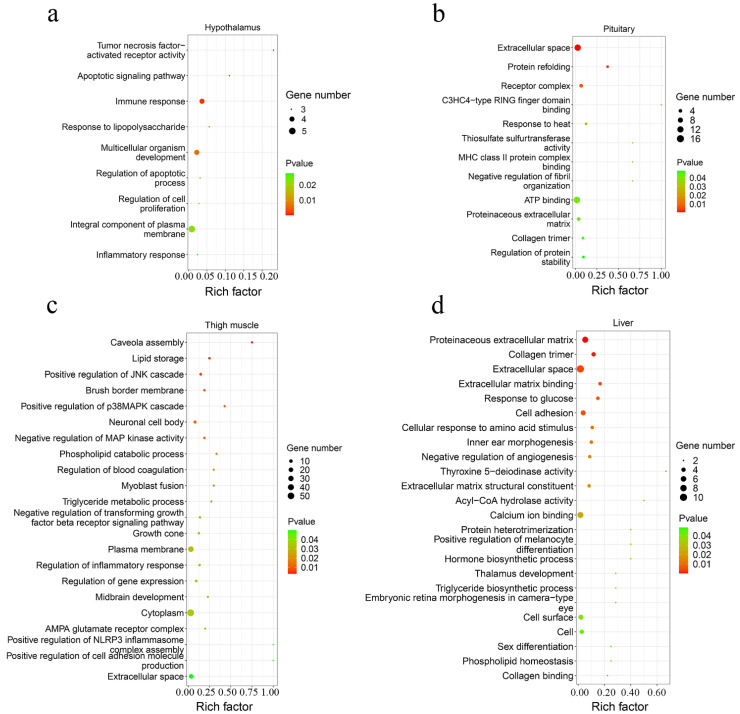
The scatter plot of GO functional enrichment. The enriched GO terms of DEGs in the hypothalamus (**a**), pituitary (**b**), thigh muscle (**c**), and liver (**d**).

**Figure 5:**
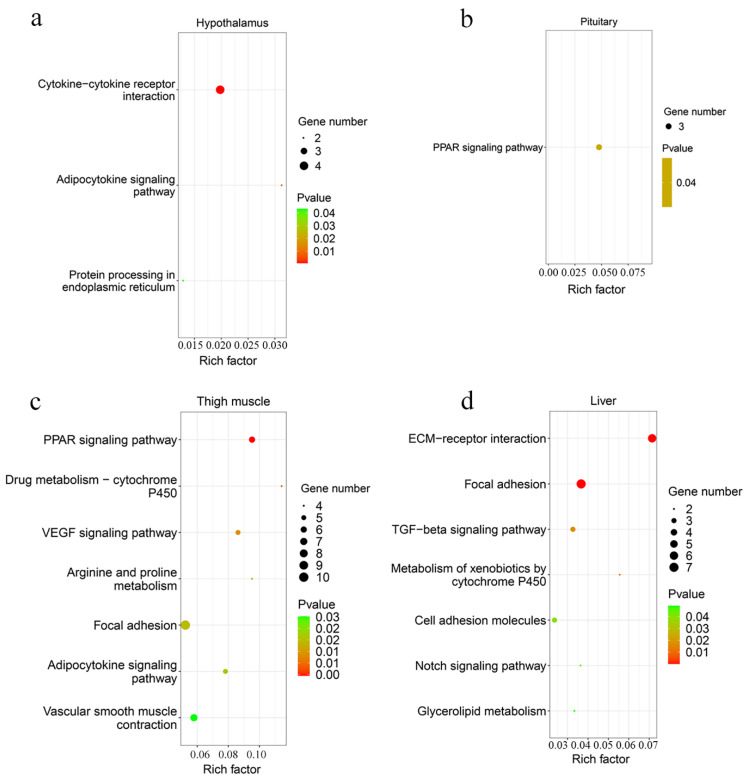
The scatter plot of KEGG pathway analysis. The enriched KEGG pathway of DEGs in the hypothalamus (**a**), pituitary (**b**), thigh muscle (**c**), and liver (**d**).

A total of 42 up-regulated and 9 down-regulated genes were identified in the hypothalamus, and 141 increased and 69 decreased genes were screened in the pituitary (Figure 3b,c, Supplementary Table S3). Among them, there were 11 common DEGs in the hypothalamus and pituitary, which included *DS cell adhesion molecule* (*DSCAM*), *peptidase inhibitor 16* (*PI16*), *leptin receptor* (*LEPR*), and others. In total, 9 and 12 Gene Oncology (GO) terms significantly enriched terms were found in the hypothalamus and pituitary, respectively (Figure 4a,b). *TNFRSF18*, *TNFRSF8*, and *TNFRSF1B* dominated almost all enriched GO items in the hypothalamus (Supplementary Table S4). Furthermore, these three genes and *LEPR* were involved in the cytokine-cytokine receptor interaction pathway, which is the prominent KEGG pathway in the hypothalamus (Figure 5a, Table 2 and Supplementary Table S5). Nevertheless, only the PPAR signaling pathway was enriched significantly in the pituitary, which included *HMGCS1*, *ACSL6*, and *PLTP* genes (Figure 5b, Table 2 and Supplementary Table S5).

#### 3.2.2. Muscle Tissues

A total of 193 up-regulated genes and 195 down-regulated genes were identified in the thigh muscle, but only 21 up-regulated genes and 10 down-regulated genes were found in breast muscle (Figure 3d,e, Supplementary Table S3). The expression of genes related to muscle development, such as *MYH1D*, *MSTN*, *MYH10*, *MYLPF*, *MYLK4*, *MYL1*, and *MYBPC1*, were altered in muscle tissues. There were 12 DEGs both in breast muscle and thigh muscle, such as *MYBPC1*, *UBB*, *MHCIY*, *SLC25A30*, *RSRP1*, *DDX59*, and *DDIT4*. The 388 DEGs of the thigh muscle were enriched significantly to 22 GO terms and 7 KEGG pathways (Figure 4c and Figure 5c). The PPAR signaling pathway had a high rich factor and showed the most significant differences, including *GK2*, *ACOX2*, *APOA1*, *ACSL1*, *FABP3*, and *CD36*. Focal adhesion and vascular smooth muscle contraction comprised the largest number of DEGs (Table 2). In addition, the functions of DEGs were also related to metabolism, such as drug metabolism-Cytochrome P450, arginine and proline metabolism, and the adipocytokine signaling pathway. Due to the fewer number of DEGs in breast muscle, we did not find significantly enriched KEGG pathway or GO terms.

#### 3.2.3. Liver Tissue

In the liver, there were 70 up-regulated and 30 down-regulated DEGs, which were mainly related to the cellular community (focal adhesion), signaling molecules and interactions (ECM-receptor interaction, cell adhesion molecules), and signal transduction (TGF-β signaling pathway, Notch signaling pathway) in liver (Figure 3f and Figure 5d, Supplementary Table S3). Almost all the genes enriched in these pathways were up-regulated in liver in the fast-growing group (Table 2). The ECM-receptor interaction pathway and focal adhesion involved the largest numbers of DEGs, which included *COL1A1*, *COL1A2*, *COL6A1*, and *COL6A3*. These four genes were also enriched in several GO terms that included an extracellular matrix structural constituent (GO:0005201), a protein heterotrimerization (GO:0070208), a cellular response to amino acid stimulus (GO:0071230), and cell adhesion (GO:0007155) (Figure 4d, Supplementary Table S4). Interestingly, the focal adhesion was a common pathway between the liver and thigh muscles. Among enrichment pathways of liver, glycerolipid metabolism and metabolism of xenobiotics by cytochrome P450 were related to metabolism.

### 3.3. Interaction Network between DEGs

**Figure 6:**
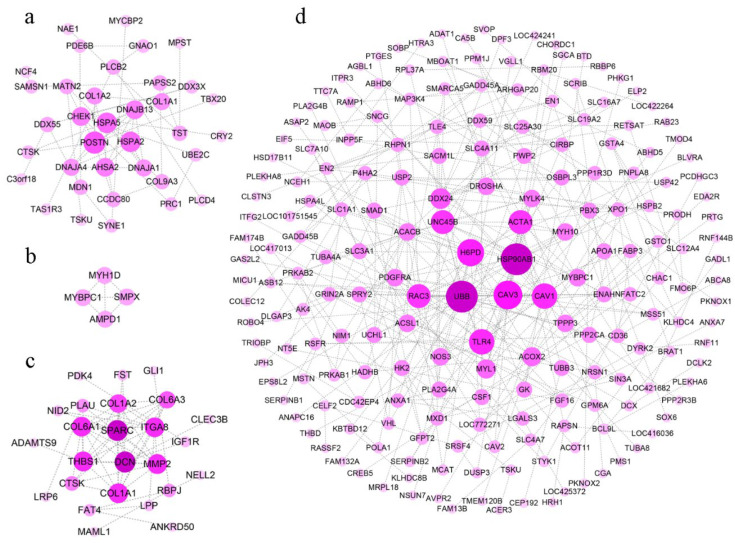
The protein-protein interaction network based on the DEGs between line S and line D. The protein-protein interaction network of DEGs in pituitary (**a**), breast muscle (**b**), thigh muscle (**c**), and liver (**d**) were analyzed and visualized by String and Cytoscape. The darker dots in the center indicate higher levels of gene interaction degree.

To clearly discover hub genes related to growth traits in a complex regulatory network, gene interactions were explored using the String database, and we constructed protein interaction networks in different tissues. DEGs in the hypothalamus failed to form regulatory networks because of a weak association in this study. Based on DEGs in the pituitary, the protein interaction network in the pituitary suggested that *POSTN*, *HSPA2*, *HSPA5*, *DNAJB13*, *AHSA2*, and *DNAJA1* occupied the most critical locations (Figure 6a).

Only *MYBPC1*, *SMPX*, *MYH1D*, and *AMPD1* were involved in the formation of the regulatory network in a few DEGs in breast muscle (Figure 6b). These genes were related to metabolic pathways and muscle contraction. In thigh muscle, abundant DEGs showed more complex regulatory networks, in which *UBB*, *CAV3*, *TLR4*, *HSP90AB1*, *H6PD*, *ACTA1*, and several genes that were expressed peculiarly in myosin (i.e., *MYH10*, *MYL1*, and *MYBPC1*) were hub genes (Figure 6c).

In the regulatory network in the liver, the hub genes (i.e., *DSN*, *ITGA8*, *THBS1*, *COL1A1*, *COL6A3*, and *COL1A2*) were engaged in ECM-receptor interaction, focal adhesion, and the TGF-β signaling pathway (Figure 6d, Table 2). These pathways were up-regulated, which indicated there was increased metabolic activity in the liver during rapid growth.

### 3.4. Validation of RNA-seq

**Figure 7:**
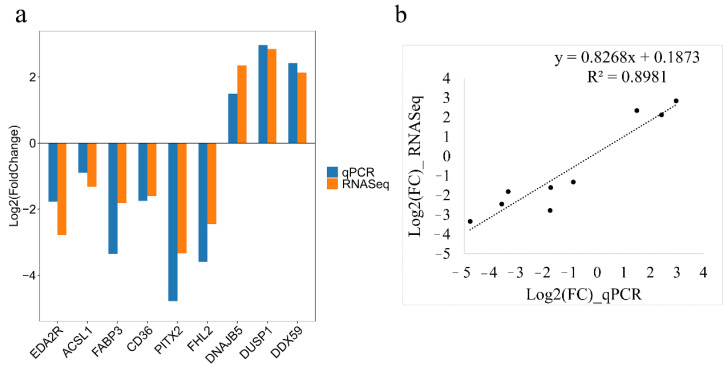
Validation of the RNA sequencing analysis data by quantitative real-time PCR (qRT-PCR) analysis. (**a**) The expression levels of differentially expressed genes were determined by qRT-PCR and RNA-Seq. Log_2_(FoldChange) < 0 represents down-regulated expression in line S and log_2_(FoldChange) > 0 represents up-regulated expression in line S. (**b**) The correlation analysis of gene expression between RNA-seq and qRT-PCR.

Nine DEGs were selected randomly to verify the accuracy of RNA-seq by the qRT-PCR method. The qRT-PCR analysis indicated decreased expression of *EDA2R*, *ACSL1*, *CD36*, *PITX2*, *FHL2*, and *FABP3* genes and increased expression of *DNAJB5*, *DUSP1*, and *DDX59* genes in the fast-growing line S of chickens, which was consistent with transcriptome results (Figure 7).

## 4. Discussion

Traditionally, the long growth period of indigenous breeds contributes to low profits in the poultry industry. In this study, distinct phenotypic differences were observed between these two lines. The body weight of the fast-growing line S after four generations of selective breeding increased 14.25% (215 g) on average compared with the slow-growing line D. Under identical feed conditions, commercial chickens showed a higher myofiber area and a decreased amount of breast muscle [17]. In the present study, myofiber area and density between the two lines in breast muscle exhibited no significant difference. High amounts of myofiber improved the weight of skeletal muscle, and it also enhanced body weight [18]. In addition, our results showed that the myofiber area of the breast muscle was significantly lower than that of the thigh muscle from both lines, which was consistent with studies of fast-growing Ross 308 broilers and slow-growing Xueshan chickens [18]. Growth rate not only affects muscle fiber density and size, but also affects meat quality, such as meat color and drop loss [19]. In our previous study, we found that the lightness, yellowness, and drip loss were elevated in breast and thigh muscles from Guangxi Partridge line S chickens, and pH and shear force were decreased in muscles from line S chickens [20]. Here, we speculated that weight differences caused by artificial selection led to a greater influence on thigh muscle, which was consistent with a higher number of DEGs and more distinct phenotypic differences in the thigh muscle.

We used transcriptome analysis of various tissues to explore the biological mechanisms of artificial selective pressure for growth rate in broilers. In summary, 51, 210, 31, 388, and 100 DEGs were found in hypothalamus, pituitary, breast muscle, thigh muscle, and liver, respectively. The results from nine DEGs detected by qRT-PCR were consistent with RNA-Seq, which proved the reliability of transcriptome sequencing results. Based on functional analysis and pathway analysis, there were differences in cell proliferation and differentiation, muscle development, metabolic processes, and signal transduction after artificial selection for growth rate.

### 4.1. Hypothalamus and Pituitary

*TNFRSF18*, *TNFRSF8*, *TNFRSF1B*, and *LEPR* genes were identified in the hypothalamus and they came in cytokine–cytokine receptor interaction signaling pathways. The TNF receptor superfamily (TNFRSF) is the receptor of the TNF superfamily (TNFSF) of cytokine-like molecules. The interactions of TNFSF ligands and TNFRSF receptors mediated signaling that was involved in survival, proliferation, and differentiation [21]. The TNF-α triggered different key steps in the insulin signaling pathway and then altered insulin sensitivity, which was important for regulation of feed intake [22,23]. Previous studies have suggested that the cytokine-cytokine receptor interaction signaling pathway played a role in upstream regulatory pathways of PPAR signaling pathways in lipid metabolism [24].

In this study, three DEGs (i.e., *HMGCS1*, *ACSL6*, and *PLTP*) and six DEGs (i.e., *GK2*, *ACOX2*, *APOA1*, *ACSL1*, *FABP3*, and *CD36*) that were found in the pituitary and thigh muscle, respectively, were involved in the PPAR signaling pathway, which functions in lipid metabolism, regulation of muscle fiber type, and energy utilization [25,26,27]. PPAR signaling is a key signaling pathway for muscle growth and regeneration, and it is dependent on activation of the PI3K-Akt-mTOR signaling axis to exert functions [28].

Growth and development of creatures are regulated by several hormones secreted by the hypothalamus and the pituitary. However, no significant change in the expression levels of hormones and their associated receptor genes were detected in the hypothalamus and pituitary except for *FST*, *TSHB*, and *LEPR*. *Follistatin* (*FST*) promoted muscle fiber formation and regulated muscle mass by inhibiting the binding activity of myostatin and its receptor, which determined increased expression in breast muscle in fast-growing exotic broilers [29,30,31]. *Thyrotropin subunit β isoform X1* (*TSHB*) regulated seasonal reproductive behavior in birds; compared with Red Junglefowl, the level of *TSHB* was higher in the pituitary of White Leghorn chickens, which indicated a contribution to domesticated traits [32]. The *LEPR* gene encoded the leptin receptor, which functioned as a crucial regulator of food intake and body weight by regulating insulin sensitivity through the JAK2 and STAT3 pathways [33,34,35,36]. Polymorphisms of the *LEPR* gene were associated with backfat thickness, intramuscular fat content and growth in pigs, and feed efficiency in chickens [37,38]. Significant alteration of these hormone receptors and pathways in the hypothalamus and the pituitary may clarify the growth difference between the two lines by regulating feed intake, insulin sensitivity, downstream muscle growth, and metabolic signaling pathways.

### 4.2. Skeletal Muscle

Several pathways for thigh muscle in the selected bred line S were associated with lipid metabolism, such as the adipocytokine signaling pathway, the PPAR signaling pathway, and glycerolipid metabolism, which included *CD36*, *PRKAB1*, *PRKAB2*, *FABP3*, and others. The membrane fatty acid transporter *CD36* is known for its role in metabolism and oxidation of fatty acids. The expression of *CD36* was enhanced to improve fatty acid transport when muscle oxidation capacity was increased [39]. *PRKAB1* and *PRKAB2* encoded the β1 and γ2 regulatory subunit of activated protein kinase (AMPK). These two genes were associated significantly with feed intake, feed conversion ratio, and body weight [40].

As expected, most of the genes critical for myogenesis were upregulated in line S with increased growth rates, such as *MHCIY*, *MYBPC1*, and *MYH1D* in breast muscle, and *MHCIY*, *MYLPF*, *MYLK4*, *MYL1*, and *ACTA1* in thigh muscle. Nevertheless, there were also genes like *MYH10*, *MYH1D*, and *MYBPC1* with lower expression levels in thigh muscle of line S. These genes were also highly connected in the protein interaction network.

Myofibers can be divided into oxidative (type I and IIA) and glycolytic fibers (type IIB) in chickens. Oxidative fibers and glycolytic fibers exhibit different contractility, glycolytic metabolism and oxidative metabolism based on mitochondrial oxidative phosphorylation [41]. In addition, the ratio of oxidative (type IIA) fibers and glycolytic fibers showed a temporal-dependent pattern and impact on pork quality [42,43]. The down-regulated *MYL1*, which is a fast muscle fiber maker [44], and the up-regulated slow-type isoform *MYBPC1*, indicated that the proportion of glycolic myofibers was increased in thigh muscle of line S chickens. This is consistent with a previous study that the size and types of myofibers between meat-type and egg-type chickens were different, and selection for growth promoted radial hypertrophy of type II muscle fibers [45]. In general, we hypothesized that selective pressure transformed myofiber types by changes in related genes and resulted further in weight variation in the fast-growing line S. In total, lipid metabolism and muscle development explained the increased growth rate and hypertrophy of muscle fiber in the selected line.

There are several other candidate genes associated with production traits. RNA-Seq showed down-regulated expression of *PITX1* and *PITX2* in thigh muscle tissue of line S. *Paired-like homeodomain transcription factor 2* (*PITX2*) and *paired-like homeodomain transcription factor 1* (*PITX1*) are important transcription factors, which were highly expressed in skeletal muscle and modulate skeletal muscle development and organ morphogenesis [46,47,48]. Multiple studies have shown that *PITX2* was correlated significantly with the growth traits and meat quality of chickens, pigs, cattle, sheep, and other animals [49,50,51,52,53,54]. Mice with over-expressed *PITX1* exhibited decreased body weight, muscle mass, muscle strength, and atrophic muscle fibers [55]. However, the causal variants of *PITX1* generated the feathered leg trait in chickens and pigeons without impairment of growth performance [56,57]. *AMP deaminase 1 isoform X1* (*AMPD1*) that was expressed specifically in skeletal muscle, was up-regulated during muscle development [58]. The *AMPD1* gene was associated with meat production and growth through backfat thickness, body weight, and inosine monophosphate acid concentrations [59,60,61]. According to our results, up-regulated *AMPD1* in breast muscle of line S indicated increased activity related to muscle development. These genes are involved in variations in lines as regulators of body weight and control of growth performance.

### 4.3. Liver

In liver, fast-growing line S focused on specific signaling pathways. Signaling molecules and interactions (i.e., ECM-receptor interaction, cell adhesion molecules), cellular community—eukaryotes (focal adhesion), and signal transduction (i.e., TGF-β signaling pathway, notch signaling pathway) suggested that the speedy development was affected by the integration of complex pathways. Focal adhesion, which connects the extracellular matrix and cytoskeleton, was an important site for the signal transduction pathway in various biological processes [62]. ECM-receptor interaction and focal adhesion were the most enriched pathways in three chicken breeds with different growth rates [4], which was consistent with this study. *COL1A1*, *COL1A2*, *COL6A1*, and *COL6A3* that encode collagen α chain are involved in ECM-receptor interaction and focal adhesion. Mutations in these four genes were associated with myopathy and osteogenesis imperfecta [63,64]. *Secreted protein acidic and cysteine rich* (*SPARC*), *thrombospondin 1* (*THBS1*), and *decorin* (*DCN*), which occupied the core of the regulatory network, are extracellular matrix-related genes. *SPARC* and *DCN* were associated with insulin resistance and obesity [65,66]. Secretion of SPARC by adipose tissue was increased by insulin and the adipokine leptin [65]. THBS1 bound to many transforming growth factors and cell surface receptors to regulate cellular adhesion, platelet aggregation, angiogenesis, and hepatic steatosis [67]. As a potential mediator of insulin resistance, THBS1 reflected the complex phenotype between obesity and metabolic syndrome or diabetes in humans [66]. DCN that was involved in cell growth and angiogenesis inhibited tumor progression and fibrosis [68].

Interestingly, almost all the genes enriched in these pathways were upregulated in the liver along with increased growth, which indicated a significant improvement in liver metabolic function in the fast-growing line S. Increased activity of signaling molecular pathways and cell community in liver affects the interactions of multiple complex pathways. This suggests appropriate changes in signaling pathways and metabolic function are required to accommodate improved growth performance.

## 5. Conclusions

The growth rate of Guangxi Partridge chickens has been improved profoundly after four generations of selective breeding for growth velocity. Transcriptome analysis was adopted to explore developmental mechanisms of growth and functional features of each tissue between two lines with different growth rates. Reduced *LEPR* expression in the hypothalamus and the pituitary reduced the inhibitory effect on food intake and weight gain through leptin signaling pathways and many neuroendocrine processes. The cytokine-cytokine receptor interaction signaling pathway in the hypothalamus, which regulated insulin sensitivity, varied the uptake and utilization of fatty acids though the PPAR signaling pathway, the adipocytokine signaling pathway, and glycerolipid metabolism. Genes that were related to myogenesis, such as *MHCIY*, *MYBPC1*, and *MYL1*, suggested that the muscle fiber type and muscle development was transformed in muscle tissue. Cellular community-related activities and pathways were improved in the liver. Collectively, our results determined the developmental dynamics in several tissues between the two lines and provide new insight into mechanisms of breeding.

## Figures and Tables

**Table 1:** Body and organ weights of line D and line S at d90.

	Line D (*n* = 10)	Line S (*n* = 10)
Body weight (kg)	1.509 ± 0.084 ***	1.724 ± 0.128 ***
Heart (g)	7.61 ± 0.96 **	8.99 ± 0.83 **
Liver (g) Lung (g) Kidney (g) Spleen (g) Testis (g)	29.56 ± 2.81 * 9.88 ± 1.97 3.20 ± 0.96 * 3.03 ± 0.77 10.90 ± 8.80	32.55 ± 2.73 * 9.42 ± 0.60 5.88 ± 2.78 * 3.65 ± 1.44 14.89 ± 7.18

Values are shown as mean ± SD. The asterisk in rows shows significant results for each indicator between two lines: * represents *p* < 0.05, ** represents *p* < 0.01, and *** represents *p* < 0.001.

**Table 2:** The significantly enriched KEGG pathway and related differential genes in each tissue.

Tissue	Term and Pathways	*p*-Value	DEGs No.	Genes	
				Up-Regulated	Down-Regulated
Hypothalamus	Cytokine–cytokine receptor interaction	6.7 × 10^−4^	4	*TNFRSF18*/*TNFRSF8* /*TNFRSF1B*	*LEPR*
	Adipocytokine signaling pathway	0.008	2	*TNFRSF1B*	*LEPR*
	Protein processing in endoplasmic reticulum	0.043	2	*DNAJA1*/*DNAJB1*	
Pituitary	PPAR signaling pathway	0.040	3	*HMGCS1*/*ACSL6*/*PLTP*	
Thigh muscle	PPAR signaling pathway	0.004	6	*GK2*/*ACOX2*	*APOA1*/*ACSL1*/*FABP3*/*CD36*
	Drug metabolism—cytochrome P450	0.010	4	*GSTA4L*	*GSTO1*/*MAOB*/*FMO3*
	VEGF signaling pathway	0.014	5	*PLA2G4B*/*RAC3*	*LOC107057170* /*NFATC2*/*PLA2G4A*
	Arginine and proline metabolism	0.020	4	*P4HA2*	*LOC107057170* /*PRODH*/*MAOB*
	Focal adhesion	0.020	10	*MYLK4*/*RAC3*/*MYLPF* /*CAV3*	*TNX*/*CAPN2* /*PDFRA*/*CAV1* /*COL4A6*/*CAV2*
	Adipocytokine signaling pathway	0.021	5	*PRKAB1*/*PRKAB2*	*ACSL1*/*ACACB*/*CD36*
	Vascular smooth muscle contraction	0.030	7	*PLA2G4B*/*RAMP1*/*MYLK4*	*PLA2G4A*/*ITPR3* /*KCNMB2*/*MYH10*
Liver	ECM-receptor interaction	7.8 × 10^−6^	6	*COL1A1*/*COL1A2*/*COL6A3* /*ITGA8*/*COL6A1*/*THBS1*	
	Focal adhesion	9.87 × 10^−5^	7	*COL1A1*/*COL1A2*/*COL6A3* /*ITGA8*/*IGF1R*/*COL6A1* /*THBS1*	
	TGF-β signaling pathway	0.017	3	*FST*/*THBS1*/*DCN*	
	Metabolism of xenobiotics by cytochrome P450	0.019	2	*LOC100859645*/*CYP1B1*	
	Cell adhesion molecules	0.039	3	*NECTIN3*/*SIGLEC1*/*ITGA8*	
	Notch signaling pathway	0.042	2	*MAML1*/*RBPJ*	
	Glycerolipid metabolism	0.049	2	*LPIN1*	*GPAM*

DEGs No. represents the number of DEGs in the enriched KEGG pathway in line S compared with line D. The up-regulated and down-regulated DEGs between line S and line D in each pathway were listed.

## Data Availability

The raw sequence data of the current study were deposited in the NCBI Sequence Read Archive (SRA), http://www.ncbi.nlm.nih.gov/bioproject/PRJNA809839 (accessed on 24 February 2022).

## Supplementary Materials

The following are available online at https://www.mdpi.com/article/10.3390/genes13050798/s1, Figure S1: The heatmap of correlations between all tissues; Figure S2: The statistical power of RNA-seq differential expression analysis; Figure S3: The heatmap of DEGs in each tissue; Table S1: Primers used for qRT-PCR; Table S2: Overall statistics and read mapping to the reference for each library; Table S3: List of DEGs in each tissue between line S and line D chickens; Table S4: List of significantly enriched GO terms of DEGs; and Table S5: List of significantly enriched KEGG pathway of DEGs.
