# Supplementary material for: Transcriptome Analysis Reveals the Differentially Expressed Genes Associated with Growth in Guangxi Partridge Chickens
Source: Genes (Basel). 2022 Apr 29;13(5):798. doi: 10.3390/genes13050798 (PMC9140345; doi:10.3390/genes13050798)
Supplement: Supplementary file 1 [file genes-13-00798-s001.zip › genes-1667907-supplementray material(Figures s1-s3, tables s1,s2).pdf]

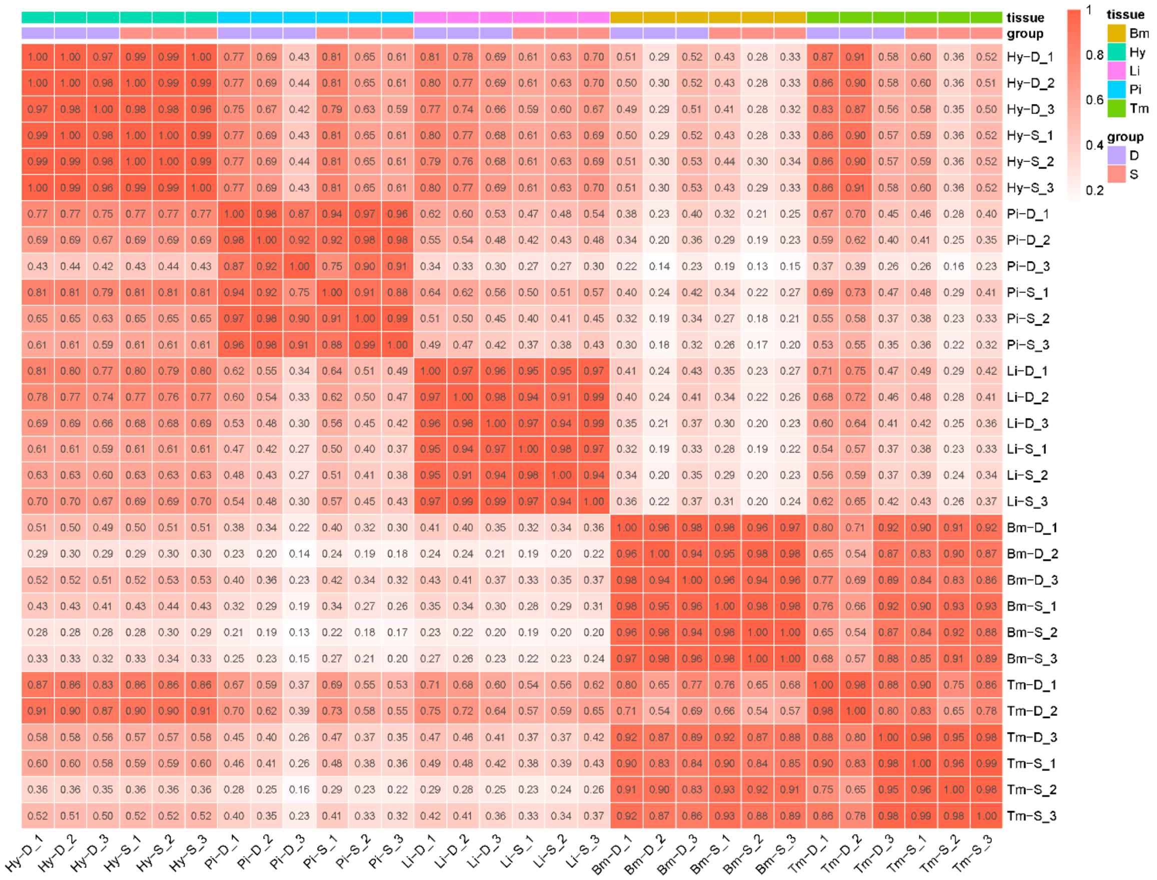

Figure S1. The heatmap of correlations between all tissues.

The abscissa and ordinate in the figure are all samples, and the color depth represents the correlation coefficient of the two samples. Hy, Hypothalamus; Pi, Pituitary; Bm, Breast muscle; Tm, Thigh muscle; Li, Liver.

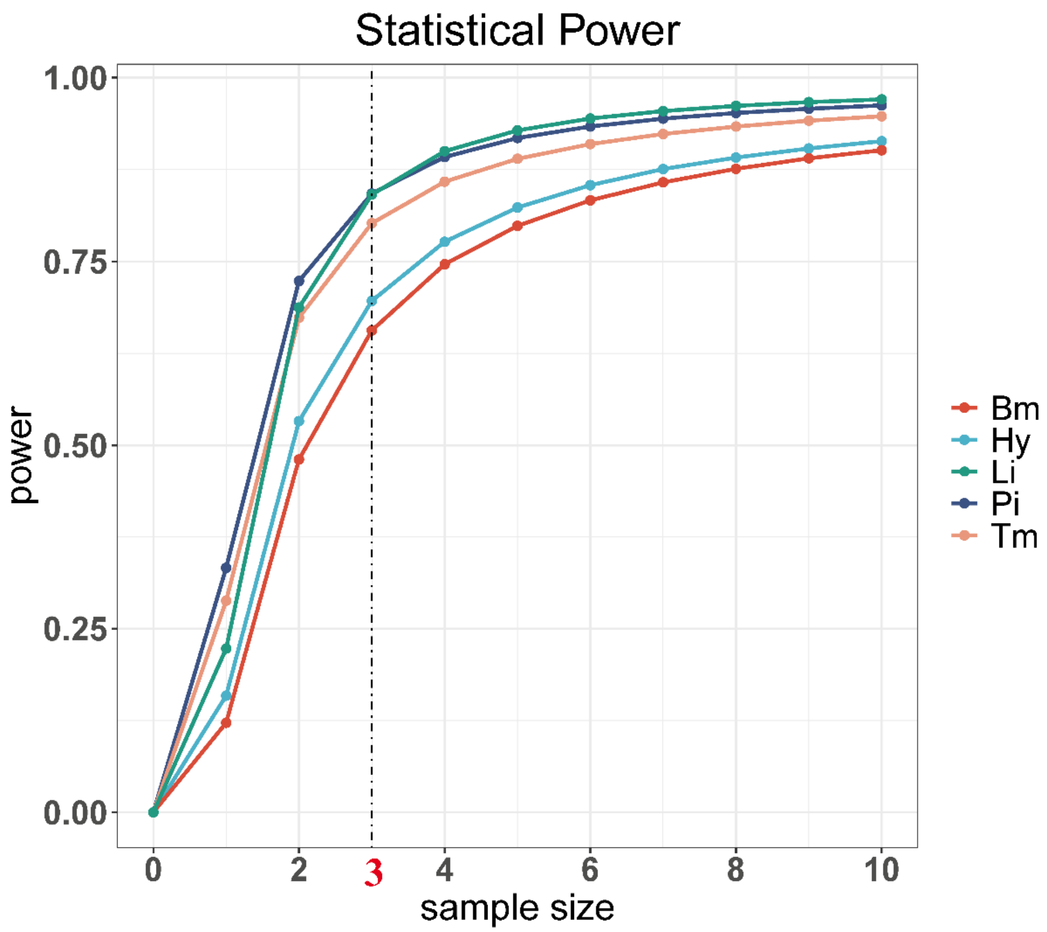

Figure S2. The statistical power of RNA-seq differential expression analysis.

The sample size of this RNA-seq was 3, and the statistical power reached 0.69, 0.84, 0.65,0.80 and 0.84 for hypothalamus, pituitary, breast muscle, thigh muscle and liver, respectively.

Hy, Hypothalamus; Pi, Pituitary; Bm, Breast muscle; Tm, Thigh muscle; Li, Liver.

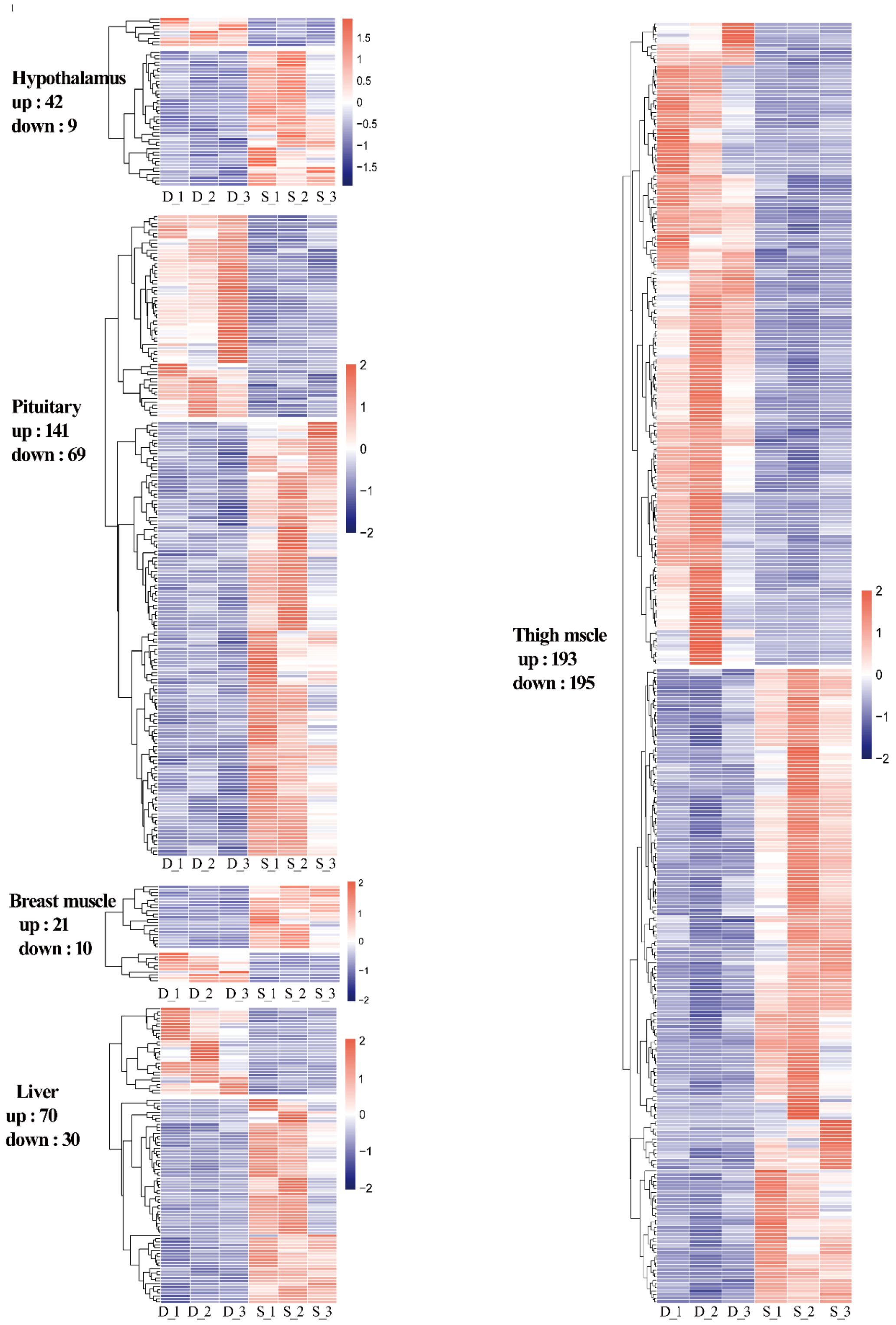

Figure S3. The heatmap of DEGs in each tissue

The heatmap showed the clustering analysis of DEGs in each tissue. Each row represents a gene and upregulation in line S is indicated in red, while downregulation in line S is shown in blue. sample ID is indicated underneath the corresponding column. The number represents the number of DEGs in each tissue. Hy, Hypothalamus; Pi, Pituitary; Bm, Breast muscle; Tm, Thigh muscle; Li, Liver.

Table S1. Primers used for qRT-PCR

Gene symbol	Primer sequence, 5'- 3'	Product Length (bp)
EDA2R	F: GCAGAACTTCTCAGGCCAAC	176
	R: TGGCTTCTCCCTCTGAAATG	
ACSL1	F: GGACAGGCGGATACATCACT	186
	R: TGCAGATACAGGTGCTGCTC	
CD36	F: CACTGCAATTTGCCAAAAGA	198
	R: TCTGCACCACACCCAGTAAC	
PITX2	F: CCTCCAAGAAGAAGCGGCAG	185
	R: CCATTTGGCTCTGCGGTTCT	
FABP3	F: ACGGTGAAGACCCATAGCAC	193
	R: TCAACTTCCCATCCTTCAGC	
FHL2	F: CAATCAGTGGTCTCGGAGGA	150
	R: TTCCACATTCAGGGCAGAGG	
DNAJB5	F: AAGCCTCACTCGCACTTCAA	150
	R: CGGCTTGATGATGTCGTTGC	
DUSP1	F: GTTTAACGAGGCGATTGACTTC	121
	R: GGTCCTCATGAGATAAGCAAGG	
DDX59	F: GTGGGTGGAAGAGCCATCTA	192
	R: GCTGTCCTTTCCACCTGAGA	

Note: F represents the forward primers and R represents the reverse primers.

Table S2. Overall statistics and read mapping to the reference for each library

Sample	Raw Reads	Clean Reads	Q20	Q30	Mapped Ratio	Exonic Rate	Intronic Rate	Intergenic Rate
Hy-D_1	55510094	55155322 (99.36%)	97.15%	92.33%	93.91%	85.26%	8.79%	5.95%
Hy-D_2	48065766	47774262 (99.39%)	97.20%	92.44%	93.81%	83.03%	10.80%	6.17%
Hy-D_3	57396248	57064130 (99.42%)	97.29%	92.63%	93.90%	83.91%	9.82%	6.28%
Hy-S_1	50780404	50480590 (99.41%)	97.10%	92.20%	93.69%	84.00%	9.69%	6.31%
Hy-S_2	47110080	46852310 (99.45%)	97.28%	92.61%	93.81%	82.85%	10.89%	6.26%
Hy-S_3	51476916	51150160 (99.37%)	97.29%	92.68%	93.61%	83.43%	10.51%	6.06%
Pi-D_1	49772804	49451248 (99.35%)	97.10%	92.32%	92.91%	82.22%	11.95%	5.83%
Pi-D_2	50272652	49938464 (99.34%)	97.03%	92.28%	92.10%	81.25%	12.17%	6.58%
Pi-D_3	52506288	52200488 (99.42%)	97.13%	92.29%	93.52%	77.97%	15.88%	6.15%
Pi-S_1	49893786	49537972 (99.29%)	96.82%	92.01%	90.86%	82.92%	8.43%	8.65%
Pi-S_2	52241282	51877382 (99.30%)	96.67%	91.43%	91.92%	83.35%	9.63%	7.02%
Pi-S_3	49361198	49026472 (99.32%)	97.26%	92.86%	91.32%	83.34%	9.89%	6.77%
Li-D_1	45262290	45014884 (99.45%)	97.53%	93.00%	95.62%	88.37%	8.64%	2.98%
Li-D_2	47930970	47671476 (99.46%)	97.69%	93.46%	95.29%	88.16%	8.84%	3.00%
Li-D_3	57938834	57667710 (99.53%)	97.65%	93.27%	95.62%	88.31%	8.72%	2.96%
Li-S_1	45789206	45552508 (99.48%)	97.59%	93.15%	95.25%	86.43%	10.06%	3.51%
Li-S_2	52019412	51747550 (99.48%)	97.67%	93.43%	95.02%	84.61%	11.57%	3.83%
Li-S_3	50541792	50274410 (99.47%)	97.38%	92.67%	95.34%	88.20%	8.83%	2.96%
Bm-D_1	55320514	55012318 (99.44%)	97.50%	93.12%	90.37%	90.36%	6.16%	3.48%
Bm-D_2	50411692	50142230 (99.47%)	97.60%	93.41%	89.68%	89.88%	6.64%	3.47%
Bm-D_3	52894472	52638184 (99.52%)	97.52%	93.14%	90.42%	90.78%	6.09%	3.13%
Bm-S_1	50708138	50472738 (99.54%)	97.60%	93.30%	90.05%	91.22%	5.76%	3.03%
Bm-S_2	51636250	51375958 (99.50%)	97.53%	93.18%	89.77%	90.37%	6.16%	3.48%
Bm-S_3	56921350	56615914 (99.46%)	97.42%	92.94%	89.39%	90.46%	6.49%	3.05%
Tm-D_1	44741890	44502332 (99.46%)	97.31%	92.56%	93.18%	90.36%	6.75%	2.90%
Tm-D_2	49948638	49672694 (99.45%)	97.43%	92.86%	94.85%	89.26%	7.61%	3.12%
Tm-D_3	51583616	51313082 (99.48%)	97.39%	92.79%	91.12%	90.64%	6.52%	2.83%
Tm-S_1	45370962	45140188 (99.49%)	97.57%	93.20%	91.25%	91.15%	6.00%	2.85%

Tm-S_2	47177656	46941784 (99.50%)	97.81%	93.81%	90.17%	91.25%	5.81%	2.95%
Tm-S_3	45297424	45075012 (99.51%)	97.62%	93.31%	91.16%	91.08%	6.10%	2.81%

Note: Q20 represents an error rate less than 0.01; Q30 represents an error rate less than 0.001.
